# Effects of Multilevel Facetectomy and Screw Density on Postoperative Changes in Spinal Rod Contour in Thoracic Adolescent Idiopathic Scoliosis Surgery

**DOI:** 10.1371/journal.pone.0161906

**Published:** 2016-08-26

**Authors:** Terufumi Kokabu, Hideki Sudo, Yuichiro Abe, Manabu Ito, Yoichi M. Ito, Norimasa Iwasaki

**Affiliations:** 1 Department of Orthopaedic Surgery, Hokkaido University Hospital, Sapporo, Hokkaido, Japan; 2 Eniwa Hospital, Eniwa, Hokkaido, Japan; 3 Department of Spine and Spinal Cord Disorders, Hokkaido Medical Center, Sapporo, Hokkaido, Japan; 4 Department of Biostatistics, Hokkaido University Graduate School of Medicine, Sapporo, Hokkaido, Japan; Harvard Medical School/BIDMC, UNITED STATES

## Abstract

Flattening of the preimplantation rod contour in the sagittal plane influences thoracic kyphosis (TK) restoration in adolescent idiopathic scoliosis (AIS) surgery. The effects of multilevel facetectomy and screw density on postoperative changes in spinal rod contour have not been documented. This study aimed to evaluate the effects of multilevel facetectomy and screw density on changes in spinal rod contour from before implantation to after surgical correction of thoracic curves in patients with AIS prospectively. The concave and convex rod shapes from patients with thoracic AIS (n = 49) were traced prior to insertion. Postoperative sagittal rod shape was determined by computed tomography. The angle of intersection of the tangents to the rod end points was measured. Multiple stepwise linear regression analysis was used to identify variables independently predictive of change in rod contour (Δθ). Average Δθ at the concave and convex side were 13.6° ± 7.5° and 4.3° ± 4.8°, respectively. The Δθ at the concave side was significantly greater than that of the convex side (*P* < 0.0001) and significantly correlated with Risser sign (*P* = 0.032), the preoperative main thoracic Cobb angle (*P* = 0.031), the preoperative TK angle (*P* = 0.012), and the number of facetectomy levels (*P* = 0.007). Furthermore, a Δθ at the concave side ≥14° significantly correlated with the postoperative TK angle (*P* = 0.003), the number of facetectomy levels (*P* = 0.021), and screw density at the concave side (*P* = 0.008). Rod deformation at the concave side suggests that corrective forces acting on that side are greater than on the convex side. Multilevel facetectomy and/or screw density at the concave side have positive effects on reducing the rod deformation that can lead to a loss of TK angle postoperatively.

## Introduction

Restoration and maintenance of the normal sagittal contour as well as coronal correction of the thoracic curve is an important surgical strategy in patients with thoracic adolescent idiopathic scoliosis (AIS), because these patients typically have a hypokyphotic thoracic spine compared with nonscoliosis patients [1]. Currently, posterior segmental pedicle screw (PS) instrumentation and fusion has become one of the most common surgical treatments. However, recent studies have reported that PS constructs to maximize scoliosis correction can cause further lordosis of the thoracic spine [2–4]. These patients exhibit a flat back, leading to progressive decompensation and sagittal imbalance [1,5]. Preservation of thoracic kyphosis (TK) is also critical to maintain lumbar lordosis after surgical treatment of AIS [1].

To overcome these issues, Ito et al. [6] and Sudo et al. [7–9] recently developed a very simple surgical technique called the simultaneous double-rod rotation technique (SDRRT) for correcting AIS. In this technique, two rods are connected to the screw heads and are simply rotated simultaneously to correct the scoliosis, while TK is maintained or improved. Moreover, hypokyphotic rod deformation is prevented with dual-rod derotation instead of single-rod derotation [6–9].

Some studies have investigated the correlation between AIS curve correction and destabilization procedures such as multilevel facetectomy [10] or the number of fixation anchors, such as PS density [11–14]. Implant rod curvature will also influence the postoperative TK. The initial shape of the rod could lead to a certain sagittal outcome. However, it has been recognized that rods bent by surgeons prior to implantation tend to flatten after surgery [15,16]. The postoperative implant rod deformation as a “spring-back” effect can alter the sagittal alignment of the spine and consequently the clinical outcome [17]. Until now, there has been no consensus on what possible factors can alter the shape of the rod. Based on the biomechanical point of view, the comprehensive effects of the surgical strategies on postoperative TK remain unknown. This study aimed to evaluate the effects of multilevel facetectomy and/or screw density on the change in the rod contour and TK in patients with thoracic AIS.

## Materials and Methods

### Patients

This study was an investigator-initiated observational cohort study conducted at a single medical center and approved by institutional review board of Hokkaido University Hospital (approval number: 014–0370). A written informed consent was obtained from all participants. Data from 49 patients (1 male, 48 female) with Lenke type 1 or type 2 AIS curves who underwent posterior thoracic curve correction between June 2009 and April 2016 were evaluated at our institution. Exclusion criteria included syndromic, neuromuscular, and congenital scoliosis and the presence of other double or triple major AIS curves, as well as thoracolumbar and lumbar AIS curves. The average age and Risser sign at surgery were 15.5 ± 2.2 years (range, 12–20) and 3.9 ± 1.1 (range, 1–5; Table 1), respectively.

**Table 1:** Disease characteristics and clinical features of the subjects.

	Mean ± standard deviation	Range
Body mass index (kg/m^2^)	18.8 ± 2.4	12.4 to 24.2
Risser sign (grade)	3.9 ± 1.1	1 to 5
Preoperative main thoracic Cobb angle (°)	59.5 ± 10.2	46 to 88
Postoperative main thoracic Cobb angle (°)	13.3 ± 7.3	1 to 36
Preoperative thoracic kyphosis angle (°)	11.7 ± 7.8	-4 to 34
Postoperative thoracic kyphosis angle (°)	21.1 ± 6.3	7 to 33
Number of vertebrae in fusion (no.)	10.8 ± 1.6	7 to 14
Number of facetectomy levels (no.)	6.5 ± 3.4	0 to 12
Screw density at concave side (no. of screws / level instrumented)	0.89 ± 0.14	0.5 to 1
Screw density at convex side (no. of screws / level instrumented)	0.80 ± 0.16	0.4 to 1

Standing long-cassette posteroanterior and lateral radiographs were evaluated for multiple parameters before and at the 2-week follow-up. Coronal and sagittal Cobb angle measurements of the main thoracic (MT) curves were obtained. The end vertebrae levels were determined on preoperative radiographs and measured on subsequent radiographs to maintain consistency for statistical comparisons [7,8]. Sagittal measurements included the TK (T5–T12) angle [7,8]. The number of facetectomy levels was counted, and screw density was expressed as the number of screws per level instrumented for each patient. In this study, the number of hooks in the instrumented level was not counted.

### Surgical Technique

Six-millimeter diameter titanium-alloy implant rods and polyaxial PSs (USS II Polyaxial, DePuy Synthes, Raynham, MA, USA) were used to correct the scoliosis deformity. All rods were prebent only at a single plane. Rods and screws were surgically implanted via the double rod rotation technique [6–9]. In this technique, two implant rods were inserted into the polyaxial screw heads. The polyaxial screw heads remained unfastened until the completion of rod rotation, allowing the rods to rotate and translate freely inside the screw head. A torque was applied to the rod-rotating device to rotate the rods simultaneously, transferring the previous curvature of the rod at the coronal plane to the sagittal plane. Additional in situ bending or other reduction maneuvers were not performed in all cases. All polyaxial screws were carried upward and medially to the concave side of the curve by the rotation of the rods, which did not exert any downward force on the vertebral body [6–9]. Both polyaxial screw heads and simultaneous double-rod rotation were key to the current technique. Frictional force at the screw–rod interface was decreased, and there was little chance of screw cut-out laterally[9]. This technique provided derotation of the apical vertebra as well as restoration of TK, leading to rib hump correction without additional costoplasty [9].

### Rod Analysis

**Fig 1:**
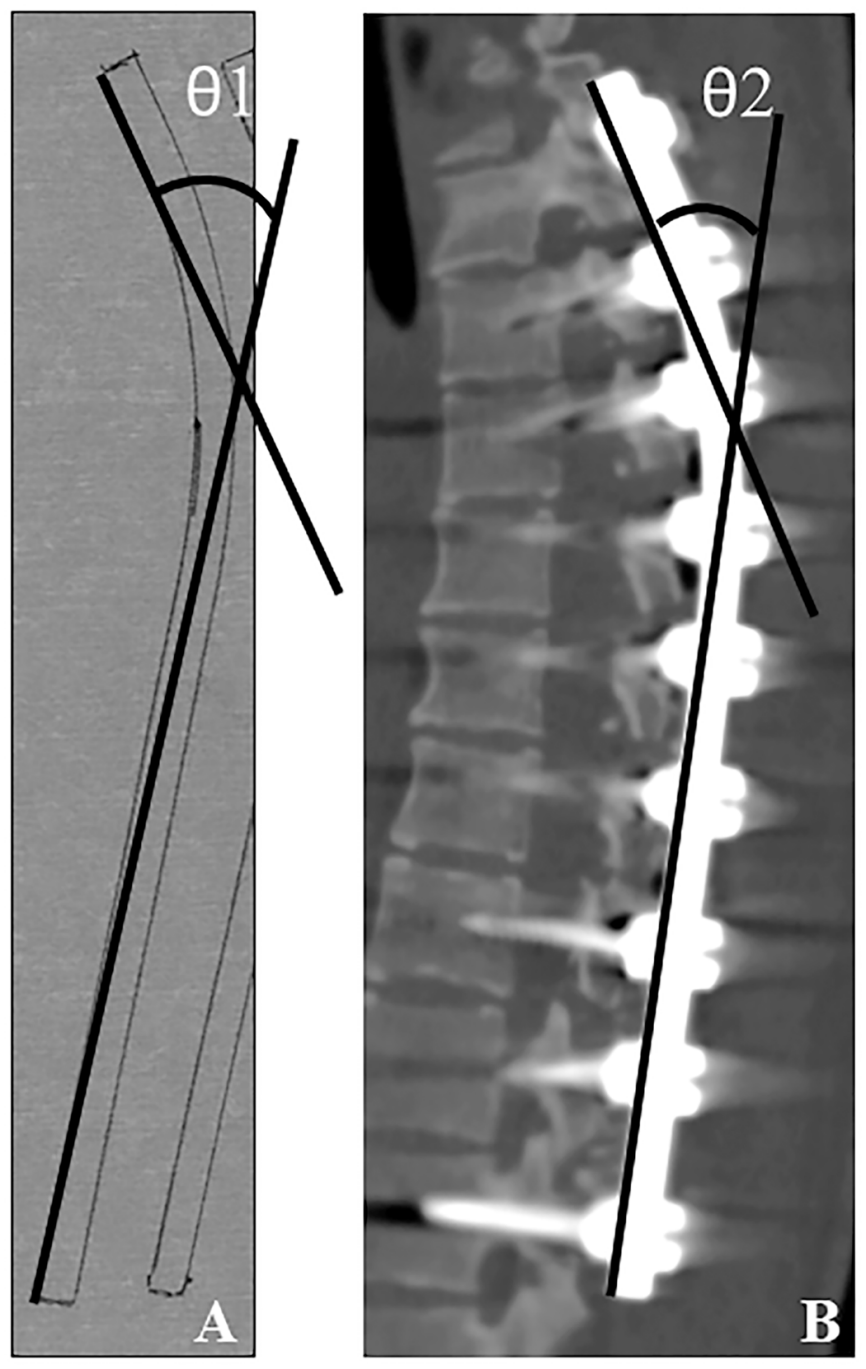
Rod angle before and after implantation. (**A**) Prior to implantation, the surgeon traced the rod shapes on paper. The angle between the proximal and distal tangential line was measured (θ1). (**B**) Postoperative implant rod geometry (θ2) was obtained after the surgical operation using computed tomography.

The implant rod angle of curvature was used to evaluate implant rod deformation. Prior to implantation, following the intraoperative contouring of the rods, the surgeon traced the rod shapes on paper [15]. The angle between the proximal and distal tangential line was measured as the rod angle before implantation (θ1) as previously described [15]. Postoperative implant rod geometry was obtained a maximum of 2 weeks after the surgical operation using computed tomography (Aquilion 64 CT scan; Toshiba Medical Systems Corporation, Tokyo, Japan). Digital Imaging and Communications in Medicine (DICOM) data were obtained to reconstruct new images by DICOM viewer software (OsiriX Imaging Software; Pixmeo Labs., Geneva, Switzerland). The reconstructed sagittal images of the implanted rods were obtained, and the angle between the proximal tangential line and the distal tangential line was measured (θ2) (Fig 1). In cases in which the rod shape had both thoracic and lumbar curvature, the distal tangential line was determined based on the inflection point. The angle of rod deformation (Δθ) was defined as the difference between θ1 and θ2 (θ1–θ2). The angles θ1, θ2, and Δθ were obtained from the rods at both the concave and convex sides.

### Statistical Analysis

Bivariate statistical analysis was performed between the change in TK (postoperative TK–preoperative TK) and the Δθ at the concave or convex side using the Wilcoxon rank sum test. Pearson’s correlation coefficient analysis was used to assess relationships between independent variables. Stepwise linear regression analysis was applied to control for possible confounding variables and to identify variables independently predictive of Δθ both at the concave and convex side. Patients’ age and disease characteristics were included in the variables: age, body mass index [weight (kg)/height(m)^2^], Risser sign, preoperative MT Cobb angle, postoperative MT Cobb angle, preoperative TK angle, postoperative TK angle, number of facetectomy levels, and screw density at both the concave and convex side. Significant multivariate predictors are reported with their respective predictive equations, including the intercept and regression coefficients (β). Model fit was assessed by using the goodness-of-fit *F* test and *R*^*2*^ statistic. Data analyses were performed using JMP statistical software for Windows (version 12; SAS, Inc., Cary, NC, USA). *P* < 0.05 was considered statistically significant. All data are expressed as mean ± standard deviation.

## Results

Disease characteristics are summarized in Table 1. On average, 10.8 ± 1.6 vertebrae were instrumented in the 49 patients. The average preoperative MT curve was 59.5° ± 10.2°. Postoperative radiographs showed an average MT curve of 13.3° ± 7.3°. Sagittal plane analysis revealed that the average preoperative TK was 11.7° ± 7.8°, which improved significantly to 21.1° ± 6.3° (*P* < 0.0001).

The preoperative θ1 and postoperative θ2 implant rod angle of curvatures at the concave and convex sides of the deformity are listed in Table 2.

**Table 2:** Implant rod angle of curvature at the concave and convex side of deformity.

	Mean ± standard deviation	Range
Preoperative rod angle (θ1) at concave side (°)	41.8 ± 7.1	22.3 to 66.5
Preoperative rod angle (θ1) at convex side (°)	38.4 ± 9.5	19.5 to 69.9
Postoperative rod angle (θ2) at concave side (°)	28.2 ± 9.1	9.2 to 48.5
Postoperative rod angle (θ2) at convex side (°)	34.1 ± 8.2	15.0 to 55.8
Rod deformation (Δθ) at concave side (°)	13.6 ± 7.5	-0.3 to 36.5
Rod deformation (Δθ) at convex side (°)	4.3 ±4.8	-6.8 to 17.8

**Fig 2:**
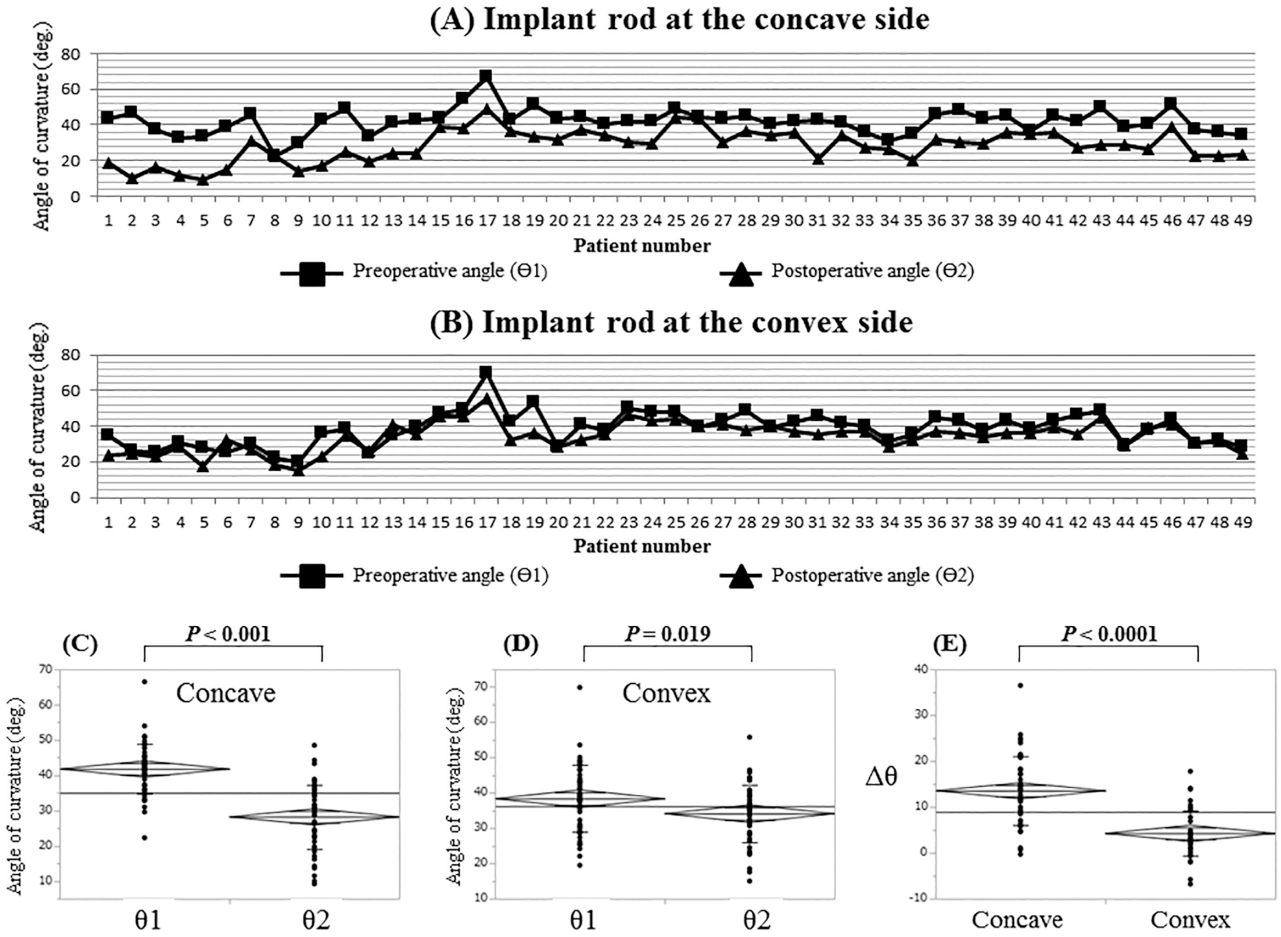
Implant rod angle of curvature at the concave and convex sides of the deformity. (**A**) θ1 and θ2 at the concave side of each patients. (**B**) θ1 and θ2 at the convex side of each patients. (**C**) Comparison between θ1 and θ2 at the concave side. (**D**) Comparison between θ1 and θ2 at the convex side. (**E**) Comparison between Δθ at the concave side and Δθ at the convex side.

The θ2 was significantly lower than the θ1 at the concave side (*P* < 0.001 at the concave side, *P* = 0.019 at the convex side, respectively). The Δθ at the concave side was significantly greater than that of the convex side (*P* < 0.0001) (Fig 2).

**Fig 3:**
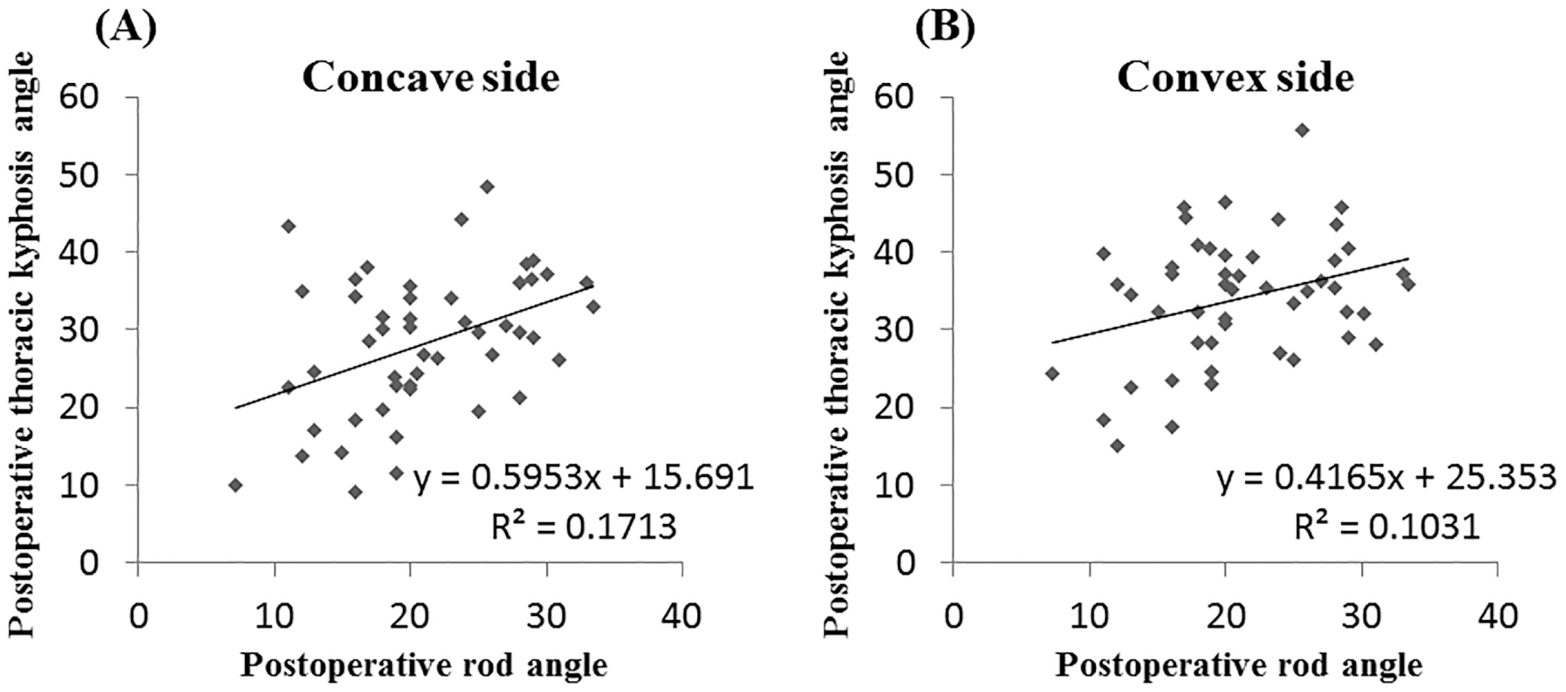
Correlation analysis between the postoperative rod angle and the thoracic kyphosis angle. (**A**) concave side. (**B**) convex side.

Postoperative TK was significantly correlated with the postoperative θ2 implant rod angle at both sides, particularly at the concave side (concave: *r* = –0.415, *P* = 0.003; convex: *r* = –0.321, *P* = 0.025, respectively) (Fig 3).

In multiple stepwise linear regression analysis, 4 variables were independent predictive factors for Δθ at the concave side: Risser sign (*P* = 0.032), the preoperative MT Cobb angle (*P* = 0.031), the preoperative TK angle (*P* = 0.012, and the number of facetectomy levels (*P* = 0.007). The model fit the data well (goodness-of-fit *F* test = 7.05, *R*^2^ = 0.50, *P* = 0.0001) (Table 3).

**Table 3:** Associations between various factors and rod deformation at the concave side (°) using multiple stepwise linear regression analysis.

	Regression Coefficient	Standard Error	95% Confidence Interval	t	Standardized β	*P*
Constant	-10.787	8.57	(-28.081, 6.509)	-1.26	-	0.215
Risser sign (grade)	1.668	0.753	(0.148, 3.188)	2.21	0.249	0.032
Preoperative main thoracic Cobb angle (°)	0.265	0.085	(0.095, 0.436)	3.14	0.362	0.031
Preoperative thoracic kypohosis angle(°)	-0.279	0.106	(-0.494, -0.064)	-2.62	-0.292	0.012
Number of facetectomy levels (no.)	-0.716	0.253	(-1.225, -0.206)	-2.83	-0.325	0.007
Screw density at convex side (no. of screws / level instrumented)	10.372	5.205	(-0.133, 20.876)	1.99	0.223	0.053

*P* < 0.05 was considered statistically significant

Conversely, for Δθ at the convex side, 3 variables emerged as predictors: the number of vertebrae in fusion (standardized β = –0.596, *P* = 0.0003), the number of facetectomy levels (standardized β = 0.578, *P* = 0.0006), and the Risser sign (standardized β = –0.292, *P* = 0.026). However, *R*^2^ was low (goodness-of-fit *F* test = 5.67, *R*^2^ = 0.34, *P* = 0.0009), indicating that only 34% of the variation in Δθ was explained by these 3 predictors.

### Subgroup Analysis

To determine whether Δθ affects postoperative TK, the total cohort was then divided into 2 groups on the basis of the mean Δθ at the concave side. The Δθ ≥ 14° group was defined by Δθ above the mean degree (13.6° ± 7.5°) at the concave side and further analyzed. The average age (n = 23) were 15.4 ± 2.1 years (range, 12–20). Disease characteristics and rod data in the group of ≥ 14° rod deformation are summarized in Table 4.

**Table 4:** Disease characteristics and rod data in the group of ≥ 14° rod deformation at the concave side.

	Mean ± standard deviation	Range
Body mass index (kg/m^2^)	18.6 ± 2.5	13.1 to 23.4
Risser sign (grade)	4.0 ± 0.9	1 to 5
Preoperative main thoracic Cobb angle (°)	61.7 ± 11.7	46 to 88
Postoperative main thoracic Cobb angle (°)	14.0 ± 6.3	1 to 27
Preoperative thoracic kyphosis angle (°)	7.6 ±5.5	-4 to 23
Postoperative thoracic kyphosis angle (°)	19.6 ± 6.0	7 to 33
Number of vertebrae in fusion (no.)	10.5 ± 1.6	7 to 13
Number of facetectomy levels (no.)	5.6 ± 3.4	0 to 11
Screw density at concave side (no. of screws / level instrumented)	0.89 ± 0.15	0.56 to 1
Screw density at convex side (no. of screws / level instrumented)	0.84 ± 0.15	0.5 to 1
Preoperative rod angle (θ1) at concave side (°)	43.4 ± 8.0	29.6 to 66.5
Preoperative rod angle (θ1) at convex side (°)	37.9 ± 11.3	19.5 to 69.9
Postoperative rod angle (θ2) at concave side (°)	23.7 ± 9.6	9.2 to 48.5
Postoperative rod angle (θ2) at convex side (°)	32.5 ± 9.3	15.0 to 55.8
Rod deformation (Δθ) at concave side (°)	19.7 ± 5.3	14.1 to 36.5
Rod deformation (Δθ) at convex side (°)	5.5 ± 6.0	-6.8 to 17.8

Pearson’s correlation coefficient analysis showed that in the group of Δθ ≥ 14°, Δθ at the concave side had significant correlation with the postoperative TK angle (*r* = −0.590, *P* = 0.003), the number of facetectomy levels (*r* = −0.479, *P* = 0.021), and screw density at the concave side (*r* = −0.537, *P* = 0.008)(Table 5).

**Table 5:** Correlation analysis between rod deformation and variable in patients with rod deformation ≥14 ° at the concave side.

	Pearson’s correlation coefficients		
Variable	Correlation coefficient	95% CI	Statistical significance
Age at surgery (yrs)	*r* = -0.017	(-0.398, 0.427)	*P* = 0.937
Body mass index (kg/m^2^)	*r* = -0.207	(-0.570, 0.225)	*P* = 0.344
Risser sign (grade)	*r* = -0.084	(-0.479, 0.340)	*P* = 0.705
Preoperative main thoracic Cobb angle (°)	*r* = 0.142	(-0.287, 0.524)	*P* = 0.518
Postoperative main thoracic Cobb angle (°)	*r* = 0.396	(-0.019, 0.695)	*P* = 0.061
Preoperative thoracic kyphosis angle (°)	*r* = -0.286	(-0.625, 0.143)	*P* = 0.186
Postoperative thoracic kyphosis angle (°)	*r* = -0.590	(-0.806, -0.235)	*P* = 0.003
Number of vertebrae in fusion (no.)	*r* = -0.324	(-0.649, 0.102)	*P* = 0.132
Number of facetectomy levels (no.)	*r* = -0.479	(-0.744, -0.083)	*P* = 0.021
Screw density at concave side (no. of screws / level instrumented)	*r* = -0.537	(-0.777, -0.160)	*P* = 0.008
Screw density at convex side (no. of screws / level instrumented)	*r* = 0.350	(-0.073, 0.666)	*P* = 0.102
Rod deformation (Δθ) at convex side (°)	*r* = 0.014	(-0.424, 0.400)	*P* = 0.948

*P* < 0.05 was considered statistically significant

## Discussion

Careful investigation of the changes in implant rod geometry is important to fully understand the biomechanics of scoliosis correction [16]. However, there have been few studies examining the relationship between rod deformation and sagittal alignment of the thoracic spine [15,16,18]. Cidambi et al. [15] documented that a significant difference was observed between pre- and postoperative rod contour, particularly for concave rods, and that the resulting deformations were likely associated with substantial *in vivo* deforming forces. Similarly, Salmingo et al. [16] reported that implant rods at the concave side were significantly deformed after surgery, whereas rods at the convex side had no significant deformation. Abe et al. [18] suggested that the corrective force during scoliosis surgery was 4 times greater at the concave side than at the convex side. The present study also showed that there was a significant positive relationship between postoperative TK and the postoperative implant rod angle of curvature, indicating that implant rod curvature influences sagittal curve correction. In addition, rod deformation at the concave side was significantly greater than that of the convex side.

Removing the facets and soft tissues between the posterior elements has been shown to allow greater distraction abilities along the length of the posterior column [1]. Destabilization of the posterior spinal segment by releasing soft tissue or facet joints could be important to prevent implant breakage or pedicle fracture during maneuver in more severe curve corrections [18]. However, it is still unclear whether these posterior releases positively affect the TK, especially with a hyphokyphotic thoracic spine [1,9]. Recently, Sudo et al. [9] documented that in patients with a hypokyphotic thoracic spine < 15°, a significant correlation was found between the change in TK and the number of facetectomy levels, indicating that multilevel facetectomy is an important factor to restore TK in patients with hypokyphotic thoracic spines. In the present study, there was a significant negative correlation between preoperative TK and rod deformation, indicating that the rod deformation was greater in patients with preoperative hypokyphotic thoracic spines. In addition, the deformation could be decreased by increasing the number of facetectomy levels.

Screw density may be also a possible factor in optimizing restoration of TK. However, the effect of implant density on sagittal plane correction and TK restoration has been reported in only a few studies, and the results have been controversial [12,14,19]. Larson et al. [12] demonstrated that decreased TK was correlated with increased screw density for Lenke type 1 and 2 curves. Conversely, Liu et al. [14] documented that higher screw density provided better TK restoration than low screw density. Recently, Sudo et al. [9] also documented that in patients with preoperative TK < 15°, a significant positive correlation was found between the change in TK and screw density, whereas no correlation was found in patients with TK ≥15°, suggesting that screw density had a positive effect on TK restoration in patients with hypokyphotic thoracic spines. Their results indicate that screw density at the concave side has an impact not only on scoliosis correction but also on TK restoration.

In the present study, in patients with rod deformation at the concave side ≥ 14°, there were significant negative correlations between rod deformation at the concave side and postoperative TK or screw density at the concave side. These results suggest that rod deformation ≥ 14° at the concave side significantly decreases postoperative TK. However, this rod deformation could be decreased by increasing screw density at the concave side. Hence, the current results biomechanically supported the results presented by Sudo et al.[9], documenting that in patients with preoperative hypokyphotic thoracic spines, increasing screw density at the concave side is important for optimizing postoperative TK.

There were limitations to this study. First, we evaluated deformity surgery with the use of titanium rods. The module of elasticity of the titanium alloy is much less than either stainless steel or cobalt chrome implants [16]. Second, we did not analyze the effects of multilevel osteotomy on the *in vivo* flexibility of the thoracic spine. We are now measuring *in vivo* force acting at the vertebrae before and after multilevel osteotomies in order to investigate the biomechanical effects of spinal flexibility provided by multilevel facet osteotomies on rod deformation. Third, resisting forces from the deformed spine might be different between males and females and this would need to be addressed in our predominantly female cohort. However, there were no effects of gender on thoracic hypokyphosis postoperatively (data not shown). Last, the relationships between rod deformation and clinical symptoms remain unclear.

## Conclusion

The present study showed that there was a significant relationship between postoperative TK and the postoperative implant rod angle of curvature. In addition, the rod at the concave side was significantly deformed after the surgical treatment. The rod deformation at the concave side suggests that corrective forces acting on that side are greater than on the convex side. Multilevel facetectomy and/or screw density at the concave side have positive effects on reducing the rod deformation that can lead to a loss of TK angle postoperatively.
